# Improving quality of care for patients with high-grade glioma and their informal caregivers: Insights from focus groups with healthcare professionals

**DOI:** 10.1093/nop/npag028

**Published:** 2026-03-31

**Authors:** Tomás Gómez Vecchio, Ingela Henoch, Ramona Schenell, Stina Nyblom, Anneli Ozanne

**Affiliations:** Institute of Health and Care Sciences, Sahlgrenska Academy, University of Gothenburg, Sweden; Department of Clinical Neuroscience, Institute of Neuroscience and Physiology, University of Gothenburg, Sweden; Institute of Health and Care Sciences, Sahlgrenska Academy, University of Gothenburg, Sweden; Institute of Health and Care Sciences, Sahlgrenska Academy, University of Gothenburg, Sweden; Administration of Eldercare and Welfare, the City of Gothenburg, Gothenburg, Sweden; Palliative Centre, Sahlgrenska University Hospital, Gothenburg, Region Västra Götaland, Sweden; Institute of Medicine, Sahlgrenska Academy, University of Gothenburg, Gothenburg, Sweden; Institute of Health and Care Sciences, Sahlgrenska Academy, University of Gothenburg, Sweden; Department of Neurology, Sahlgrenska University Hospital, Gothenburg, Region Västra Götaland, Sweden

**Keywords:** attitude of health personnel, glioblastoma, informal caregivers, palliative care, supportive care

## Abstract

**Background:**

There is a concerning prevalence of unmet needs affecting both patients with high-grade glioma and their informal caregivers. By exploring the experiences and attitudes of healthcare professionals (HCPs) in regional and municipal services, this study aims to identify barriers and enablers in improving the quality of care for patients with high-grade glioma and their informal caregivers through the disease trajectory.

**Methods:**

A qualitative approach using focus group interviews was employed, involving HCPs across healthcare levels. The interviews, conducted physically and digitally from November 2024 to March 2025, followed semi-structured guides. The inclusion criteria were HCPs with direct experience in caring for patients with high-grade glioma or those who were in contact with their informal caregivers. The data was analyzed using inductive qualitative content analysis.

**Results:**

A total of 12 interviews were conducted with 44 HCPs. The sample included 35 (79%) participants from 4 regional hospitals and 9 (21%) participants from municipal healthcare services. Two categories were derived from the data: *Organization and structural decisions* and *Interactions and interpersonal dynamics*. These categories illustrate advantages and limitations in the local healthcare system together with interpersonal dynamics and mental attitudes as expressed by the HCPs interviewed.

**Conclusions:**

The study underscores the need for collaboration and better communication strategies across healthcare services, together with comprehensive patient and informal caregiver education on the potential future needs associated with the patients’ health trajectory. Also, the study shows that a better understanding of the consequences of cognitive impairments are critical steps towards enhancing care effectiveness.

Key Points

- HCPs emphasized the need for flexible, person-centered care from diagnosis to end-of-life.
- Barriers include poor interprofessional coordination and limited informal caregiver support.
- Enablers involve proactive symptom monitoring and holistic care strategies.

Importance of the Study

This study contributes to existing research by offering a system‑level perspective grounded in healthcare professionals’ experiences across both regional and municipal services. It highlights organizational and coordination challenges that influence care delivery for patients with high‑grade glioma and their informal caregivers. The findings provide actionable insights by identifying critical barriers and enablers that may inform future strategies to improve the quality of care.

Malignant brain tumors in adults are incurable.^1^ The most common type is glioma, with median overall survival ranging from 1 to more than 10 years depending on the glioma subtype and tumor grade.^2-4^ The tumor and its treatment affect the health-related quality of life of both patients and their informal caregivers, with a steady decline over time in both groups.^5-7^ Scientific reports indicate that supportive care should be tailored to individual family preferences and adapted to their changing needs as the disease progresses.^8-10^ Supportive care teams represent the integration of palliative care specialists within multidisciplinary teams.^11^^,^^12^ Furthermore, integrated care across settings, from hospital to home, often requires specialized palliative care.^8^^,^^13^ Thus, this also generates the need to optimize the balance between general and specialized palliative care.^14^ As in other incurable diseases, supportive care for patients with glioma applies to the whole disease trajectory, from diagnosis to death also including emotional and practical support for families, extending beyond the patient’s death.^15^^,^^16^ Throughout the disease trajectory, patients and their informal caregivers interact with healthcare professionals (HCPs) across multiple care settings which can result in discrepancies in access to healthcare information regarding ongoing planned, or alternative treatment options.^17^

Although several opportunities to improve the quality of care for patients with glioma have been reported in the scientific literature,^18^ disparities have been observed in how patients with malignant brain tumor utilize specialized palliative care.^19^ Furthermore, both patients and their informal caregivers have been found to partly experience insufficient support throughout the disease trajectory.^8^^,^^20-22^ To achieve further insight into the quality of care, the present study explores the perspectives of HCPs. This study is part of a larger collaborative research project aimed at improving the quality of care for patients with high-grade glioma and their informal caregivers.^6^^,^^8^^,^^23^^,^^24^ This study aims to explore the experiences of HCPs across different levels of care to identify barriers and enablers to improve the quality of care for patients with high-grade glioma and their informal caregivers through the disease trajectory.

## Methods and Analysis

### Design

The study builds on the collective knowledge from previous projects where the health-related quality of life, life experiences and healthcare supporting needs of patients with high-grade glioma and their informal caregivers have been explored.^6^^,^^8^^,^^23^^,^^24^ The current study has a qualitative design. Since the existing theory and research literature on the phenomenon is limited, we chose inductive qualitative content analysis as our methodology to systematically describe the content of the gathered material.^25^

### Researchers’ Preconceptions

All researchers have research experience focusing on populations of patients with glioma. AO has clinical neuro-oncological expertise in the care of patients with brain tumors. IH, SN, and RS have clinical expertise in general and specialized palliative care of patients with brain tumor. TGV and RS have working experience in municipal care settings. AO, IH, RS, and SN have experience in qualitative research methodological approaches.

### Participants and Settings

Participants were HCPs from regional hospitals and municipal healthcare, along with professionals from municipal social services assessor offices (hereinafter included in “HCPs” unless otherwise explicitly stated), in a region with a population of approximately 2 million inhabitants.^26^ Professionals without first-hand experience of working with patients with high-grade glioma or their informal caregivers were excluded.

Sweden’s tax-funded universal healthcare system is decentralized, leading to geographical variation in service organization and delivery. While national authorities set policies and oversee the system, the 21 regions are self-governing authorities responsible for financing and providing both primary and specialized healthcare, including hospital services. The 290 municipalities are also self-governing authorities, responsible for municipal home healthcare and healthcare provided in residential facilities for older people and persons with disabilities.^27^^,^^28^ In Sweden, palliative care specialists are not fully integrated into multidisciplinary teams. Instead, functions like those of supportive care teams are typically delivered through collaborations among contact nurses, psychosocial services, multidisciplinary neuro-oncology teams, and municipal services. Consequently, recruitment targeted HCPs from both inpatient and outpatient settings in neuro-oncology and specialized palliative care at the 5 largest regional hospitals, as well as from municipal healthcare services in the 2 largest and one medium-sized municipality, aiming to capture geographical variation.

Recruitment was conducted by email or telephone from November 2024 to March 2025. The inclusion criteria were HCPs with direct experience in caring for patients with high-grade glioma or those who were in contact with their informal caregivers. Participants were contacted via our clinical networks or, when no prior connection existed, directly through unit managers. Based on the number of units approached and assuming inclusion of at least 2 participants per unit, a minimum of 30 participants was anticipated. The ethical approval further allowed flexibility to recruit up to 50 participants should this be warranted to obtain richer and more comprehensive data. Invitations were distributed to selected units, which in turn identified and nominated staff members who met the inclusion criteria and were willing to participate. Inclusion was completed when sufficient information power was deemed to have been reached.^29^

### Data Collection

Data was collected through focus groups, paired interviews, and individual interviews. Before the sessions, participants were provided with concise project information and a one‑sentence popular‑science summary to establish a common baseline understanding.^6^^,^^8^^,^^24^^,^^30^ The interviews were guided by a semi-structured interview protocol comprising 7 overarching themes, developed based on previous studies (available in the Supplementary Material).

Interviews were conducted in person or online according to participants’ preferences, digitally recorded, and transcribed verbatim. Each session lasted a maximum of 2 hours. Demographic data including professional background and workplace were also collected.

### Data Analysis

Data, a single dataset consisting of all interviews, was assessed using the software NVivo 14 (2023 released by Lumivero, Denver, United States). No Artificial Intelligent (AI) software or automatic analysis tools were used for data analysis. The dataset was analyzed using inductive qualitative content analysis.^25^ Focus was placed on the manifest content (ie, staying close to the text). To achieve a deeper understanding and an overall sense of the material, the dataset was first read several times. The text was then divided into meaning units, from which codes were created and then sorted into subcategories and categories. The analysis was conducted through multiple iterations with focus shifting between the whole and its parts. With each stage of the analysis, the level of abstraction increased, while the degree of interpretation was deliberately kept low, although some interpretation was inherent in the process. This iterative process was accompanied by group discussions until consensus among the analysts (TGV and AO) and all co-authors was achieved. Data analysis then progressed using these categories and subcategories, which formed the basis for mapping the findings.^25^ A description of the Swedish healthcare system was used as framework for the mapping.^28^ Methodological rigor was supported through iterative team-based coding, repeated consensus discussions among analysts, and systematic application of inductive qualitative content analysis, while maintaining close engagement with the manifest content.

### Ethical Considerations

The study has been approved by the Swedish Ethics Review Authority (Dnr 2021-04040; Dnr 2024-08096-02) and it was conducted in accordance with the Declaration of Helsinki.^31^ All participants received written and verbal information about the study and provided written informed consent. Participation in the focus groups was voluntary. The dataset was pseudo-anonymized. To preserve the confidentiality of the participants, quotes and other references to the data are presented with pseudonyms referring to their professional background and healthcare level.

## Results

### Sample Characteristics

We approached 12 specialized regional healthcare units and 6 municipal healthcare and social services assessor units. The study included participants from regional neurology, oncology and geriatric clinics, regional cancer rehabilitation centers, regional palliative care teams, municipal nursing homes, municipal home healthcare, and municipal social service assessor offices. In total, 9 focus groups (with 7, 6, 5, 4, 4, 4, 3, 3, and 3 participants), 2 paired interviews, and one individual interview were conducted, totaling 44 participants overall: 35 (79%) HCPs from 4 regional hospitals and 9 (21%) HCPs from 2 municipalities. Each focus group consisted of participants from the same clinical unit or municipal services, reflecting existing daily working teams. Two paired interviews and the individual interview were carried out when scheduling constraints prevented participants from joining the planned focus groups, thereby enabling the inclusion of their perspectives without modifying the interview guide. Demographic data are provided in Table 1. Three regional units (ie, oncology and palliative care) and 3 units from municipal services (ie, nursing homes, home healthcare, and services oriented towards informal caregivers) declined participation.

**Table 1.** Participants’ characteristics

Variable	Study sample (*n* = 44)
Age, mean (SD)	49 (10)
Female, no (%)	42 (95)
Experience working with patients with malignant brain tumors, no (%)	
0 to 5 years	11 (25)
6 to 10 years	18 (41)
More than 10 years	15 (34)
Place of work, no (%)	
Hospital in- and out-patient care^a^	
Neurology clinic	18 (41)
Cancer rehabilitation center	3 (7)
Geriatric clinic	3 (7)
Oncology clinic	2 (5)
Hospital-based home care^b^	
Specialized palliative resource and mobile teams	9 (20)
Municipal healthcare services^c^	
Municipal nursing homes and home healthcare	6 (13)
Municipal social services assessor offices	3 (7)
Profession, no (%)	
Nurse/specialist nurse (specialist in neurological care, oncological care, geriatric care, and palliative care)	18 (41)
Social worker	10 (23)
Physician (specialists in neurology, oncology, and palliative medicine)	6 (14)
Physiotherapist	3 (7)
Occupational therapist	3 (7)
Neuropsychologist	2 (4)
Nutritionist	1 (2)
Speech therapist	1 (2)
Employed in executive and/or coordination roles, no (%)	5 (11)

a Distributed across 4 hospitals.

b Distributed across 2 hospitals.

c Distributed across 2 municipalities.

Teams varied in size and professional background across participating units, reflecting differences in service provision. While most teams were directly involved in patient care, some municipal participants belonged to needs‑assessment teams working within health‑services administration. Registered nurses formed the core of most teams, which relied on regular collegiate meetings and interdisciplinary discussions. Details on team composition and present working methods are presented in Table 2.

**Table 2.** Team composition and present working methods.

Municipal level	Regional level
The team composition at municipal care services variated between municipalities. The core teams were comprised of nurses, with the addition of social workers and occupational therapists in different municipality sections. These teams usually collaborated with regional PRT^a^ to provide comprehensive palliative care, emphasizing close patient interaction and communication between teams.	Teams at regional facilities were often composed of several specialists working collaboratively to provide comprehensive patient care. These teams often included social workers, nurses, neuropsychologists, and physicians. Other specialists such as physiotherapists, speech therapists and nutritionists were also represented. Each member had a specific role contributing to the overall care plan.
Teams at municipal care services utilized various resources like daily routines, meetings, and discussions to address patient needs effectively while acknowledging the complexity of managing diverse professional interactions and resource allocation.	Before initiating treatment, the team collaborated to gather necessary information from patients, informal caregivers and previous healthcare contacts. These teams often hold regular team meetings discussing each patient’s case, planning treatments, aiming for consistency in care enclosing medical and psychosocial needs.

a Specialized palliative resource team.

### Findings From the Analysis

**Figure 1.**
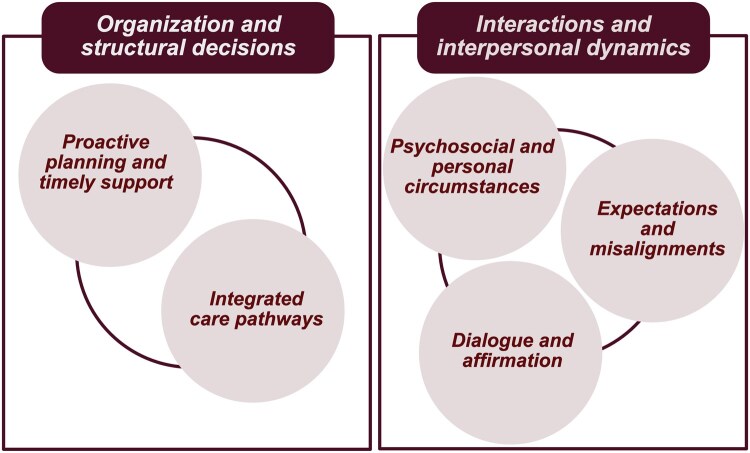
Categories and subcategories that emerged through inductive qualitative content analysis. The 2 main categories are “organization and structural decisions,” which describe barriers and enablers within the healthcare system, and “interactions and interpersonal dynamics,” which capture interpersonal processes and attitudes influencing care delivery.

The findings are organized into categories and subcategories, these are described in the following sections and illustrated in Figure 1. A mapping of the barriers and enablers identified is presented at the end of the Results section and in Table 3. Illustrative quotes appear throughout the text, while a more comprehensive set is provided in the Supplementary Material.

**Table 3.** Barriers and enablers to improving the quality of care for patients with high-grade glioma and their informal caregivers

Stakeholders	Barriers	Enablers
*National healthcarepolicymakers*	Lack of compatible record systems equally accessible to HCPs in regional and municipal services across healthcare settings (inadequate flow of sensitive information).	Recognized importance in national guidelines for addressing informal caregiver burden comprehensively including psychosocial needs. Recognized importance in national guidelines for collaboration of multi-professional teams.
*Regional and municipal healthcare policymakers*	Lack of coordination among specialized units and lack of coordination among healthcare levels (inadequate process and routines). Lack of a professional forum to discuss patient cases across healthcare levels. Limited access to specialized palliative care.	Recognized importance in regional and municipal guidelines for implementing national healthcare programs in the regional medical guidelines.
*Healthcare professionals in regional and municipal services*	Difficulties preparing informal caregivers for home caring and end-of-life scenarios. Difficulties dealing with patients facing cognitive decline. Varied non-structured approached to case management. Difficulties in generating a sense of care continuity. Lack of unified opinion to rely on patient associations and support groups. Lack of knowledge on how to deal with different cultural backgrounds and unusual spiritual experiences.	Flexible and adaptive work focused on disease progression. Clear communication among team members. Positive attitude towards repeating information to enhance understanding. Proactive approach to symptom monitoring and assessment. Professional experience in creating an environment of emotional safety, confidence, and security. Willingness to improve the quality of care.
*Patient and informal caregivers*	Inadequate illness insight and prognosis awareness. Maladaptive coping strategies to manage stress and fear. Misaligned expectations between patients and their informal caregivers. Misconceptions regarding home and palliative care.	Willingness to cope and help.

#### Organization and structural decisions

The category *Organization and structural decisions* arose from the subcategories *Proactive planning and timely support* and *Integrated care pathways*. It highlights the complexities in managing care across different settings, the challenges in transitioning between specialized units, and the need for proactive planning, clear communication, and support systems to maintain continuity of care across healthcare settings. These findings reflect several of the organizational barriers and enablers (Table 3).

##### Proactive Planning and Timely Support

Proactive planning and timely support were emphasized by raising the value of flexible, adaptive work focused on disease progression. Patients’ autonomy and privacy seemed to be managed through clear communication. Responsibilities were rarely formalized, HCPs relied on personal knowledge, local resources, and peer support among team colleagues. In specialized care, diagnostic expertise in combination with the patients and informal caregivers needs shaped practice, while in municipal home care the patient and informal caregivers’ personal needs were more in focus. Case management (eg, planning, coordination and evaluation of healthcare measures) often appeared driven by immediate needs, with HCPs describing varied, non-structured approaches.

*“An internal structure, that it’s not possible to, it’s not possible to formalize it, or it’s possible, of course, but we don’t work like that, to formalize it into a manual.” (Social worker, regional healthcare)*

Proactive planning and timely support were considered essential for managing changes in symptoms. At the same time, difficulties emerged in detecting such changes and implementing appropriate interventions, depending on where in the care trajectory the patient was situated. Contact nurses from the tumor teams played a central role, facilitating communication with formal and informal caregivers across the entire continuum of care. Initially, attention was directed primarily toward somatic symptoms, later shifting to cognitive, psychosocial, and family-related issues. Cognitive symptoms were mainly associated with speech problems, while aspects such as memory and executive functions were often overlooked. This, in turn, delayed the implementation of measures to support families facing these challenges. Over time, attention to the psychosocial situation expanded to include informal caregivers, whose burden was assessed alongside recognition of strained relationships and other unsustainable home dynamics. Although specialized teams often had a proactive approach, challenges such as time management and coordination between different care units remained unsolved. HCPs in both regional and municipal care emphasized the need for efficient communication to ensure timely support, effective symptom management and a sense of security, while also acknowledging the emotional strain of striving to be sufficient and to provide adequate care. Special attention was given to families with minors as relatives, where early and timely support from hospital social workers was considered crucial and collaboration with other units was maintained as the disease progressed.

*“(…) available care at the right time. That, that is a big challenge. Um, and maybe above all because (…) it is difficult to find a balance in how sick someone allows themselves to be or how sick one wants to look.” (Specialist nurse, regional healthcare)*

The gradual introduction of municipal home healthcare was emphasized, requiring proactive planning and clear communication to prevent caregiver overload. Ensuring anticipatory support without overstraining resources, such as preparing necessary medical equipment in advance, was also highlighted. Referrals to specialized palliative care were described as need-based but often delayed due to patient preferences or structural barriers such as limited resources and poor coordination between specialized units. Both regional and municipal HCPs emphasized the benefits of early access to municipal home healthcare but noted that support was initiated too late, largely due to deficiencies in referral procedures and coordination with regional services. Municipal HCPs described that early advance care planning was often hindered by limited contact with specialized units. They further highlighted the need for timely social services support, multilingual communication to ensure accessibility, and prompt alerts to detect changes in patients’ health or social conditions at home. End-of-life and post-death planning were complicated by rapid disease progression and cognitive decline, particularly concerning legal and financial matters.

*"I would probably start that way, from the patient’s perspective, (…) meet the patient, see this patient you have in front of you, what their needs are, and try to use our resources." (Nurse, municipal healthcare)*

##### Integrated Care Pathways

Most participants from specialized units emphasized the importance of adhering to regional medical guidelines and the national care program for patients with tumors of the brain, spinal cord and meninges. They expressed concern about care transitions and handovers, indicating that integration was only partly achieved. HCPs described that while some patients remained connected with the neurology tumor team during oncologic treatment, others were described to feel disconnected, risking unmet needs. Post-treatment coordination was considered essential, as fatigue and cognitive problems could hinder patients from seeking help. Social workers and contact nurses were crucial for ensuring communication and continuity of care, supported by improved documentation.

The main challenges concerned achieving seamless transitions between neurology, oncology, and specialized palliative care, while addressing patient needs, misconceptions, and post-treatment communication. HCPs noted that these transitions were often emotionally difficult for patients and informal caregivers, and that reluctance to engage with palliative care complicated decision making. Uncertainty about when to end oncologic therapy, loss of contact with the neurology clinic, and patients’ limited readiness or understanding about palliative care, further hindered timely transitions.

*“In general, you could say that we have pretty poor collaboration with other units and clinics, even if we in our team would like to see that it wasn’t like that, partly towards the department but also towards the oncologist and so on, and also with continuity or availability or collaboration with the municipality, that’s not the case either, we don’t have any good routine for that or anything.” (Specialist nurse, regional healthcare)*

Additional barriers to integrated care were related to municipal home healthcare, including insufficient patient education, limited communication with specialized units, and poor coordination across care levels. Incompatible record systems further hindered information sharing and continuity of care. Regional and municipal HCPs highlighted administrative barriers and resource shortages as key obstacles to care transitions.

*“So, I can say that, sometimes it can vary a lot if the referral is designed for home care or for the PRT (Palliative Resource Team from the regional hospital), because they have much more information in their referral than what we get (…)” (Nurse, municipal healthcare)*

#### Interactions and interpersonal dynamics

The category *Interactions and interpersonal dynamics* arose from the 3 subcategories *Psychosocial and personal circumstances, Expectations and misalignments,* and *Dialogue and affirmation*. These subcategories highlight the complexities of care delivery and the importance of clear communication and supportive relationships, despite barriers among patients and informal caregivers, such as fear, misconceptions, or resistance to change. These dynamics reflect key relational barriers and enablers (Table 3).

##### Psychosocial and Personal Circumstances

HCPs described inconsistencies in patients and informal caregivers understanding of the disease, its psychological impact, and implications for independence and vulnerability. They noted that patients and informal caregivers struggled to maintain normality and often lacked knowledge about prognosis and expected survival. Differing family perceptions caused stress and fear, influencing care strategies. HCPs described that patients and informal caregivers sometimes hesitated between preserving autonomy and accepting home healthcare, delaying support. Fear of losing independence, daily pressures, and unexpected informal caregiving tasks risked caregiver burnout. Personality changes further complicated communication, and informal caregivers were advised to limit detailed discussions when these interactions caused emotional strain, to protect caregivers’ well-being and maintain caregiving capacity.

*“Well, I think that many patients may not really know the prognosis that well when we meet them. And then I don’t know how thorough we are in telling them how it will develop.” (Specialist physician, regional healthcare)*

##### Expectations and Misalignments

HCPs found that both the expectations of patients and informal caregivers, as well as misunderstandings between them or with healthcare providers, could complicate the care situation. Trust, honest communication about prognosis, emotional support, collaborative and flexible care tailored to individual needs, and autonomy-respecting discussions were strongly emphasized. HCPs noted that patients and informal caregivers sometimes resisted change, highlighting the need for clear communication and attention to cognitive abilities for informed decisions. A recurring misconception was viewing palliative care solely as end-of-life care rather than symptom management and supportive care. Some informal caregivers’ challenges exceeded the responsibilities of a particular healthcare unit. These situations required coordinated efforts to align informal caregiver preparedness, patient understanding, and communication across healthcare levels. Municipal social services were seen as largely patient-focused, leaving informal caregivers feeling excluded, though early involvement was seen as key to improving preparedness and preventing overload.

*“Too many people don’t know what we can do and what we have the opportunity to offer, (…) so that we still need to help them and tell them what there is and how we work, what we can offer.” (Nurse, municipal healthcare)*

HCPs identified critical issues between patients and their informal caregivers, such as psychological violence, lack of preparedness, misaligned expectations, multiple caregiving roles, and conflicts over accepting municipal homecare. Informal caregivers were seen as vulnerable to emotional stress and overburdened by household duties, affecting mental health and family dynamics. Limited preparation for end-of-life care further strained wellbeing, while differing expectations between the patient and their informal caregiver, often rooted in limited disease knowledge or compromised decision-making abilities due to cognitive decline, added tension and complicated HCPs roles.

*"No. You want to be able to manage on your own for as long as possible. And it often becomes difficult for relatives, because the relatives must step into that role. You may want to be a partner and not a caregiver, so that is also a difficulty (…)" (Other members of the multidisciplinary team, regional healthcare)*

##### Dialogue and Affirmation

Meeting patients and informal caregivers as individuals based on their unique circumstances was central. HCPs emphasized repeating information to ensure understanding when the person was ready. They stressed the importance of considering each person’s cultural background, which was particularly highlighted in palliative care team discussions. Understanding personal perspectives on life, illness, and death and adapting communication accordingly were considered essential. Respect, empathy, and attention to individual preferences, such as privacy or avoiding emotional discussions, were crucial for supportive relationships. Language barriers highlighted the need for inclusive communication, and participants sought to create safe spaces where patients and caregivers could express themselves without fear of judgment. Municipal HCPs demonstrated a person-centered approach, focusing more on the person’s needs than solely on the disease or diagnostic label.

*“Absolutely, and you have to say that we, I don’t know much about this part of your culture, can you tell me, is there anything that I need to know that is important or something like that.” (Other members of the multidisciplinary team, regional healthcare)*

Views on digital care tools and information access varied. While helpful for communication, they were not adapted to cognitive impairment and were difficult for informal caregivers to access. HCPs noted an overload of written information and held mixed views on support groups, helpful for reducing isolation for some, but emotionally challenging for others. Rapid disease progression was seen as a barrier to sustaining such groups.

Interactions with patients and informal caregivers were described as conversations blending case management with supportive dialogue, often fragmented in time and content. Fatigue, mistrust or a focus on talking rather than listening were seen as causes of interrupted dialogue. HCPs aimed to be affirming and adaptive to promote emotional safety and open communication without fear of judgement. Municipal healthcare faced challenges in home-based end-of-life decisions due to families’ differing comfort levels, underscoring the need for early, well-documented planning.

*"We talk a lot. We have conversations, conversation after conversation. It is very important then, for us, to be able to talk and build trust, uh, so that when the day comes, they all feel, everyone, both in the family and also outside the family, feel safe with us and with the care we provide." (Specialist nurse, regional healthcare)*

#### Mapping of barriers and enablers to improve the quality of care

Across the categories and subcategories the analysis revealed these key barriers: inadequate flow of sensitive information across care levels, fragmented transition and limited inter-unit coordination, unstructured approaches to case management, fragmented knowledge on patient needs, inadequate prognosis awareness, and misaligned expectations. Key enablers comprised: the presence of national guidelines promoting collaboration and a holistic view, ongoing implementation of these guidelines in regional and municipal practice, a person-centered approach, and extensive professional experience across units.

## Discussion

This study explored HCPs’ experiences across healthcare levels to identify barriers and enablers to improve quality of care for patients with high-grade glioma and their informal caregivers through the disease trajectory. HCPs described challenges in meeting patients’ and informal caregivers’ needs across the continuum of care, emphasizing flexible, person-centered approaches in the lack of rigid working structures. Proactive monitoring of patients’ somatic, psychosocial, and cognitive symptoms was crucial, alongside extending psychosocial assessment to informal caregivers. A lack of proactive approaches, timely interventions and clear communication about changing care plans was identified as a key challenge. It also appeared that concurrent access to cancer-directed and palliative care was often limited. Additionally, many determinants of care quality operate at organizational or policy levels beyond individual control.

The main barriers gravitate around building trust, aligning expectations, and strengthening illness insight and prognosis awareness. HCPs in our study faced difficulties preparing patients and informal caregivers for home care and end-of-life care, potentially leading to compromised patient autonomy, psychological stress from caregiving roles and possible caregiver burnout. These findings are in line with reports from the scientific literature where HCPs focused on engaging in meaningful exchanges with patients and informal caregivers.^32^^,^^33^ We found that poor coordination among HCPs exacerbated these challenges. Strengthened communication between specialized teams and municipal services is needed to ensure timely information sharing. Similar findings in studies on chronic disease care emphasize that interprofessional communication is essential to maintain continuity and prevent information gaps.^34^^,^^35^ Also, there seems to be an overflow of written information accessible to patients and informal caregivers. While digital care tools create the image of accessibility (regardless of time or place) there are barriers for patients affected by cognitive decline and for informal caregivers lacking the right to access patients’ health-related information. These findings go in line with concerns regarding access to information in patients with glioblastoma, previously raised by studies highlighting discrepancies in treatment information and patients’ experiences and expectations of treatment.^36^ Also, mirroring the recently highlighted need for consideration of the amount, detail and timing of the information offered to patients.^37^

Moreover, a lack of alignment between regional and municipal HCPs, and between patients’ and informal caregivers’ goals and expectations appeared linked to different understandings of the disease. This misalignment may stem from incomplete patient education not fully addressed by any of the teams. These findings align with previous studies on prognostic awareness and disease understanding in patients with malignant glioma.^38^^,^^39^

Patients and informal caregivers often rely on sticking to normalcy and to minimize the impact of cancer on their daily lives. A strategy that was found effective in other studies focusing on young patients with more indolent brain tumors.^40^ In our study focusing on adult patients with malignant gliomas, HCPs raised contrasting opinions about this strategy. These opinions might be founded in differential clinical prognosis associated to different gliomas tumors.^41^

Other barriers, such as limited cultural competence and difficulties in addressing unfamiliar patient experiences aligned with previous research showing poor cultural understanding among HCPs,^42^^,^^43^ challenges in interpreting spiritual end-of-life experiences,^44^ and difficulties addressing spiritual concerns in patients with brain tumors or advanced cancer.^45^^,^^46^

In sum, these barriers together with geographical disparities in resource allocation and the absence of a legal framework for sharing patient and informal caregiver information across healthcare levels, impact the quality of care. Our findings aligned with studies on inequalities in access to primary and specialized care and with research highlighting the often neglected impact of social determinants of health in short and long-term outcomes.^47–49^

Among the main enablers we found a willingness to improve the quality of care for patients with high-grade glioma and their informal caregivers. HCPs proactive approach to symptom monitoring was central to preventing future health issues, despite the lack of a systematic assessment structure. These findings align with studies on interval scan experiences and fear of recurrence in glioma,^50^^,^^51^ symptom experiences in acute or chronic illness,^52^ and differing attitudes toward symptom monitoring among patients and informal caregivers.^53^

Additionally, HCPs experience in creating an atmosphere of emotional safety, confidence, and security might also facilitate a further development of trust and alliance most need to educate patients and their informal caregivers about the impact of the disease and its progression. These findings mirror the conclusions of previous studies where the needs and preferences of patients with high-grade glioma and their informal caregivers were explored.^51^^,^^54^^,^^55^

Also, the recognized importance of addressing informal caregiver burden comprehensively, considering both their willingness to care and personal well-being might aid in broadening the scope of healthcare in patients with high-grade glioma. The focus on informal caregivers has gained momentum in the scientific literature, as seen in studies examining how informal caregiver navigate interactions with HCPs in the context of chronic illness,^56^ or studies focusing on short- and long-term outcomes in informal caregivers of neuro-oncology patients.^57^^,^^58^

Future efforts should focus on improving understanding of cognitive impairment, especially in relation to the resources and needs of patients and their informal caregivers, and on developing strategies to enhance communication. Emphasis is also needed on systemic reforms that shift healthcare from viewing the patient as the sole unit of care toward a holistic approach that includes relatives and informal caregivers. Our findings also raise the need for shared tools to define care goals, communication and decisions taken, together with patient and informal caregivers’ preferences. Such tools may strengthen collaboration, clarify roles and promote person-centered care. Similar conclusions have been reported in comparable studies.^59–61^

However, developing and implementing these ideas might face practical hurdles like resource limitations, lack of institutional support promoting knowledge transfer among healthcare units and levels, lack of legal frame to share relevant patient and informal caregiver sensitive information across healthcare levels, and the lack of an active forum ensuring that all stakeholders are on board discussing potential changes in healthcare policy. We are inclined to believe that collaboration between patient groups, healthcare providers and policymakers is mandatory to effectively address these issues.

### Strengths and Limitations

The study’s strength lies on its diverse participant base, covering different healthcare settings and professional backgrounds relevant for patients with high-grade glioma and their informal caregivers. This diversity enriches the understanding of healthcare experiences by capturing a wide range of scenarios. Though, the ideas and opinions of the HCPs that were unable to participate remain unknown. A limitation is that HCPs in municipal homecare usually did not distinguish between patients with high- and low-grade glioma tumors, which may affect how they view their own roles and strategies, and risks obscuring important differences. Also, unit‑based focus groups may have restricted openness and limited the exploration of cross‑team dynamics. However, this structure was selected for practical scheduling reasons and ensured that participants shared common workflows and responsibilities.

## Conclusion

The study underscores the need for systemic changes in healthcare delivery to improve the quality of care for patients with high-grade glioma and their informal caregivers. Emphasis should be placed on collaboration and better communication strategies across healthcare services, together with comprehensive patient and informal caregiver education on the potential future needs associated to the patients’ health trajectory. Also, a better understanding of the consequences of cognitive impairments are critical steps toward addressing the identified barriers and enhancing care effectiveness.

## Supplementary Material

npag028_Supplementary_Data

## Data Availability

The original data generated in the course of the study are not publicly available due to ethical and privacy considerations related to participant confidentiality. To safeguard participant identities, full transcripts and raw qualitative data cannot be shared openly. However, de-identified excerpts relevant to the analysis may be provided upon reasonable request to the corresponding author, subject to review and approval by the Swedish Ethics Review Authority.

## References

[1] Weller M , van den Bent M, Preusser M, et al EANO guidelines on the diagnosis and treatment of diffuse gliomas of adulthood. Nat Rev Clin Oncol. 2021;18:170-186. 10.1038/s41571-020-00447-z 33293629 PMC7904519

[2] Fekete B , Werlenius K, Tisell M, et al What predicts survival in glioblastoma? A population-based study of changes in clinical management and outcome. Front Surg. 2023;10:1249366. 10.3389/fsurg.2023.1249366 37711136 PMC10498299

[3] Carstam L , Corell A, Smits A, et al WHO grade loses its prognostic value in molecularly defined diffuse Lower-Grade gliomas. Front Oncol. 2021;11:803975. 10.3389/fonc.2021.803975 35083156 PMC8785215

[4] Price M , Ballard C, Benedetti J, et al CBTRUS statistical report: primary brain and other central nervous system tumors diagnosed in the United States in 2017-2021. Neuro Oncol. 2024;26:vi1-vi85. 10.1093/neuonc/noae145 39371035 PMC11456825

[5] Gomez Vecchio T , Ryden I, Ozanne A, et al Global health status and fatigue score in isocitrate dehydrogenase-mutant diffuse glioma grades 2 and 3: a longitudinal population-based study from surgery to 12-month follow-up. Neurooncol Pract. 2024;11:347-357. 10.1093/nop/npae017 38737607 PMC11085849

[6] Stahl P , Henoch I, Smits A, Rydenhag B, Ozanne A. Quality of life in patients with glioblastoma and their relatives. Acta Neuro Scand. 2022;146:82-91. 10.1111/ane.13625 PMC9324166 35470866

[7] Drewes C , Sagberg LM, Jakola AS, Solheim O. Perioperative and postoperative quality of life in patients with Glioma-A longitudinal cohort study. World Neurosurg. 2018; 117: e465-e474. 10.1016/j.wneu.2018.06.052 29920391

[8] Stahl P , Henoch I, Rydenhag B, Smits A, Ozanne A. Living with glioblastoma - the need for integrated support based on experiences of chaos, loss of autonomy, and isolation in both patients and their relatives. Support Care Cancer. 2024;32:599. 10.1007/s00520-024-08801-y 39167224 PMC11339176

[9] Gómez Vecchio T , Corell A, Buvarp D, Rydén I, Smits A, Jakola AS. Classification of adverse events following surgery in patients with diffuse lower-grade gliomas. Front Oncol. 2021;11:792878. 10.3389/fonc.2021.792878 34993147 PMC8724913

[10] Ryden I , Carstam L, Gulati S, et al Return to work following diagnosis of low-grade glioma: a nationwide matched cohort study. Neurology. 2020;95:e856-e866. 10.1212/WNL.0000000000009982 32540938 PMC7605502

[11] Okon II , Osama M, Akpan A, et al The evolving role of palliative care in older people with glioblastoma. World Neurosurg. 2024;192:140-149. 10.1016/j.wneu.2024.09.125 39362596

[12] Harrison DJ , Wu E, Singh R, et al Primary and specialist palliative care in neurosurgery: a narrative review and bibliometric analysis of glioblastoma and stroke. World Neurosurg. 2023;180:e250-e257. 10.1016/j.wneu.2023.09.048 37739173

[13] Walbert T , Schultz L, Mikkelsen T, Snyder JM, Phillips J, Fortunato JT. Prospective assessment of end-of-life symptoms and quality of life in patients with high-grade glioma. Neurooncol Pract. 2024;11:733-739. 10.1093/nop/npae056 39554791 PMC11567736

[14] Crooms RC , Nnemnbeng JF, Taylor JW, Goldstein NE, Gorbenko K, Vickrey BG. Clinician perspectives on integrating neuro-oncology and palliative care for patients with high-grade glioma. Neurooncol Pract. 2024;11:404-412. 10.1093/nop/npae022 39006519 PMC11241354

[15] Piil K , Nordentoft S, Larsen A, Jarden M. Bereaved caregivers of patients with high-grade glioma: a systematic review. BMJ Support Palliat Care. 2019;9:26-33. 10.1136/bmjspcare-2017-001386 29363550

[16] Pace A , Dirven L, Koekkoek JAF, et al European association for Neuro-Oncology (EANO) guidelines for palliative care in adults with glioma. Lancet Oncol. 2017;18:e330-e340. 10.1016/S1470-2045(17)30345-5 28593859

[17] Boele F , Rosenlund L, Nordentoft S, et al Inequalities in access to neuro-oncology supportive care and rehabilitation: a survey of healthcare professionals’ perspectives. Neurooncol Pract. 2024;11:484-493. 10.1093/nop/npae023 39006521 PMC11241368

[18] Wassef CE , Cain SA, Drummond KJ. Changing practice to improve quality of life in glioma. J Neurosurg. 2024;141:1270-1280. 10.3171/2024.2.JNS221799 38728762

[19] Ozanne A , Ohlen J, Nyblom S, Jakola AS, Smits A, Larsdotter C. Disparities in end-of-life care and place of death in people with malignant brain tumors-A Swedish registry study. Neurooncol Pract. 2025;12:511-519. 10.1093/nop/npae113 40487587 PMC12137219

[20] Faris MM , Dhillon HM, Campbell R, BRAINS Program Group, et al Unmet needs in people with high-grade glioma: defining criteria for stepped care intervention. JNCI Cancer Spectr. 2024;8:1-7. 10.1093/jncics/pkae034 PMC11218915 38730547

[21] Pointon L , Grant R, Peoples S, et al Unmet needs and wish for support of family caregivers of primary brain tumor patients. Neurooncol Pract. 2023;10:271-280. 10.1093/nop/npac099 37188166 PMC10180375

[22] Schubart JR , Kinzie MB, Farace E. Caring for the brain tumor patient: family caregiver burden and unmet needs. Neuro Oncol. 2008;10:61-72. 10.1215/15228517-2007-040 17993635 PMC2600839

[23] Fekete B , Werlenius K, Carén H, et al The Gothenburg population-based glioblastoma research database: Methodological aspects and potential impact. Neurooncol Neurosurg. 2019;2:4-6. 10.15761/NNS.1000123

[24] Stahl P , Fekete B, Henoch I, et al Health-related quality of life and emotional well-being in patients with glioblastoma and their relatives. J Neurooncol. 2020;149:347-356. 10.1007/s11060-020-03614-5 32909116 PMC7541353

[25] Elo S , Kyngas H. The qualitative content analysis process. J Adv Nurs. 2008;62:107-115. 10.1111/j.1365-2648.2007.04569.x 18352969

[26] Statistics Sweden. Population in counties and municipalities. 2023; https://www.scb.se/en/.

[27] Janlov N , Blume S, Glenngard AH, Hanspers K, Anell A, Sweden MS. Health system review. Health Syst Transit. 2023;25:1-236. https://eurohealthobservatory.who.int/publications/i/sweden-health-system-review-2023 38230685

[28] Ludvigsson JF , Bergman D, Lundgren CI, et al The healthcare system in Sweden. Eur J Epidemiol. 2025;40:563-579. 10.1007/s10654-025-01226-9 40383868 PMC12170770

[29] Malterud K , Siersma VD, Guassora AD. Sample size in qualitative interview studies: Guided by information power. Qual Health Res. 2016;26:1753-1760. 10.1177/1049732315617444 26613970

[30] Stahl P , Henoch I, Schenell R, Rydenhag B, Smits A, Ozanne A. Support interventions for patients with primary high-grade brain tumours and their relatives: a scoping review. Eur J Oncol Nurs. 2026;81:103145. 10.1016/j.ejon.2026.103145 41687207

[31] World Medical Association. World Medical Association declaration of Helsinki: ethical principles for medical research involving human subjects. JAMA. 2013;310:2191-2194. 10.1001/jama.2013.281053 24141714

[32] Jani JA , Cowan D, Ouonkap L, et al Missing the message to brain tumor patients: a 2023 twitter analysis among patients, informal caregivers, and healthcare professionals in glioblastoma multiforme. J Neurooncol. 2025;172:579-586. 10.1007/s11060-025-04948-8 39899179

[33] McDougall E , Nowak AK, Dhillon HM, Breen LJ, Piil K, Halkett GKB. What is this brain’s story?” Healthcare professionals’ perspectives on managing brain tumor-related personality and behavior changes. Neuro-Oncol Pract. 2025;12:437-447. 10.1093/nop/npaf007 PMC12137208 40487585

[34] Ljungholm L , Klinga C, Edin-Liljegren A, Ekstedt M. What matters in care continuity on the chronic care trajectory for patients and family carers?—a conceptual model. J Clin Nurs. 2022;31:1327-1338. 10.1111/jocn.15989 34351651

[35] Ljungholm L , Edin-Liljegren A, Ekstedt M, Klinga C. What is needed for continuity of care and how can we achieve it?—perceptions among multiprofessionals on the chronic care trajectory. BMC Health Serv Res. 2022;22:686. 10.1186/s12913-022-08023-0 35606787 PMC9125858

[36] Fernandez S , Short SC, Boele F. Glioblastoma patient and caregiver perspectives of treatment Side-Effects and information provision. J Patient Exp. 2025;12:23743735251331770. 10.1177/23743735251331770 40290735 PMC12032473

[37] Malmstrom A , Akesson L, Milos P, et al “Do I want to know it all?” a qualitative study of glioma patients’ perspectives on receiving information about their diagnosis and prognosis. Support Care Cancer. 2021;29:3339-3346. 10.1007/s00520-020-05846-7 33125538 PMC8062391

[38] Walsh LE , Polacek LC, Panageas K, et al Coping with glioblastoma: prognostic communication and prognostic understanding among patients with recurrent glioblastoma, caregivers, and oncologists. J Neurooncol. 2022;158:69-79. 10.1007/s11060-022-04010-x 35437688 PMC10022487

[39] Forst DA , Quain K, Landay SL, et al Perceptions of prognosis and goal of treatment in patients with malignant gliomas and their caregivers. Neurooncol Pract. 2020;7:490-497. 10.1093/nop/npaa021 33014389 PMC7516113

[40] Burgers VWG , van den Bent MJ, Rietjens JAC, et al "Double awareness"-adolescents and young adults coping with an uncertain or poor cancer prognosis: a qualitative study. Front Psychol. 2022;13:1026090. 10.3389/fpsyg.2022.1026090 36591063 PMC9795247

[41] Devaraja K , Daniels M, Tsang DS, et al Navigating life with high-grade glioma: experiences and needs of adolescents and young adults. Cancer Med. 2025;14:e70867. 10.1002/cam4.70867 40197708 PMC11976453

[42] Wahlstrom E , Landerdahl Stridsberg S, Larsson C, Stier J. School health professionals’ understanding of culture: a scoping review. BMJ Open. 2025;15:e100689. 10.1136/bmjopen-2025-100689 PMC12306337 40713056

[43] Lundberg E , Ozanne A, Dellenborg L, Ohlen J, Enstedt D. Navigating complexity: Spiritual care discourses among Swedish palliative care professionals. J Relig Health. 2025;64:2337-2361. 10.1007/s10943-024-02106-4 39162774 PMC12133967

[44] Nyblom S , Arnby M, Molander U, Benkel I. End-of-life experiences (ELEs) of spiritual nature are reported directly by patients receiving palliative care in a highly secular country: a qualitative study. Am J Hosp Palliat Care. 2021;38:1106-1111. 10.1177/1049909120969133 33111551

[45] Best M , Butow P, Olver I. Why do We find it so hard to discuss spirituality? A qualitative exploration of attitudinal barriers. J Clin Med. 2016;5:77. 10.3390/jcm5090077 27598212 PMC5039480

[46] Volz D , Grabenweger R, Best MC, et al Exploring spirituality in everyday neuro-oncology practice - nurses’ and physicians’ spiritual care toolbox. Neurooncol Pract. 2025;12:520-530. 10.1093/nop/npae120 40487572 PMC12137210

[47] Doty MM , Tikkanen RS, FitzGerald M, Fields K, Williams RD. 2nd. Income-related inequality in affordability and access to primary care In eleven High-Income countries. Health Aff (Millwood). 2021;40:113-120. 10.1377/hlthaff.2020.01566 33296228

[48] Kim SR , Kim HY, Nho JH, Ko E, Moon KS, Jung TY. Relationship among symptoms, resilience, post-traumatic growth, and quality of life in patients with glioma. Eur J Oncol Nurs. 2020;48:101830. 10.1016/j.ejon.2020.101830 32971413

[49] Sable-Smith A , Arnett KR, Nowels MA, Colborn K, Lum HD, Nowels D. Interactions with the healthcare system influence advance care planning activities: results from a representative survey in 11 developed countries. Fam Pract. 2018;35:307-311. 10.1093/fampra/cmx113 29140508 PMC5965096

[50] Boele FW , Rudkin SE, Absolom K, Latchford G, Short SC, Booth TC. The experience of interval scans for adults living with primary malignant brain tumors. Support Care Cancer. 2023;31:356. 10.1007/s00520-023-07818-z 37243744 PMC10221741

[51] Sorensen von Essen H , Dahl Steffensen K, Rom Poulsen F, Piil K. It’s like living with a ticking time bomb-a qualitative study about patients’ and their families’ experiences related to the recurrence of a high-grade glioma. Neurooncol Pract. 2025;12:723-731. 10.1093/nop/npaf026 40814430 PMC12349762

[52] Missel M , Andersen LK, Corvinius C, et al Understanding symptoms in the lives of adult patients with acute or chronic illness: a phenomenological study of patient experiences. Int J Qual Stud Health Well-Being. 2025;20:2534871. 10.1080/17482631.2025.2534871 40736357 PMC12312141

[53] Boele FW , van Uden-Kraan CF, Hilverda K, et al Attitudes and preferences toward monitoring symptoms, distress, and quality of life in glioma patients and their informal caregivers. Support Care Cancer. 2016;24:3011-3022. 10.1007/s00520-016-3112-7 26879825 PMC4877415

[54] Piil K , Jakobsen J, Christensen KB, et al Needs and preferences among patients with high-grade glioma and their caregivers—a longitudinal mixed methods study. Eur J Cancer Care (Engl). 2018;27:e12806. 10.1111/ecc.12806 29314470

[55] Halkett GKB , Lobb EA, Shaw T, et al Do carer’s levels of unmet needs change over time when caring for patients diagnosed with high-grade glioma and how are these needs correlated with distress? Support Care Cancer. 2018;26:275-286. 10.1007/s00520-017-3846-x 28808797

[56] Missel M , Kurita GP, Lindblad MKE, et al Navigating the healthcare system with chronic illness: a qualitative study of caregiver experiences. Scand J Caring Sci. 2025;39:e70094. 10.1111/scs.70094 40772519 PMC12329780

[57] Meer S , Buckle P, Miller R, et al End-of-life care experiences and long-term outcomes of bereaved neuro-oncology caregivers: a cross-sectional survey. Palliat Med. 2025;39:814-826. 10.1177/02692163251344164 40515572 PMC12227823

[58] Lion KM , Jamieson A, Billin A, Jones S, Pinkham MB, Ownsworth T. It was never about me’: a qualitative inquiry into the experiences of psychological support and perceived support needs of family caregivers of people with high-grade glioma. Palliat Med. 2024;38:874-883. 10.1177/02692163241261211 38916277

[59] Philip J , Collins A, Brand CA, et al Health care professionals’ perspectives of living and dying with primary malignant glioma: Implications for a unique cancer trajectory. Palliat Support Care. 2015;13:1519-1527. 10.1017/S1478951513000576 24138726

[60] Philip J , Collins A, Brand C, et al A proposed framework of supportive and palliative care for people with high-grade glioma. Neuro Oncol. 2018;20:391-399. 10.1093/neuonc/nox140 29016886 PMC5817948

[61] Garcia Fox R , Chukwueke UN, Sannes T, et al Glioma resource outreach with support: a program to identify and initiate supportive care interventions for unmet needs among adult lower-grade glioma patients. Neurooncol Pract. 2025;12:87-99. 10.1093/nop/npae065 39917752 PMC11798613

