# Supplementary material for: Improving quality of care for patients with high-grade glioma and their informal caregivers: Insights from focus groups with healthcare professionals
Source: Neurooncol Pract. 2026 Mar 31;13(4):694–704. doi: 10.1093/nop/npag028 (PMC13365267; doi:10.1093/nop/npag028)
Supplement: npag028_Supplementary_Data [file npag028_supplementary_data.docx]

**Supplementary material**

**Improving quality of care for patients with high-grade glioma and their informal caregivers: insights from focus groups with healthcare professionals**

1. **Semi-structured interview guide**

Overarching themes and directly raised questions within each theme:

I – Accessible Care at the Right Time

How can healthcare become more accessible?

How can we facilitate contact with the appropriate healthcare professionals?

How can collaboration be improved to ensure rapid support throughout the disease trajectory? (from both specialized care teams and municipal care)

How can municipal care contribute to providing prompt interventions that enhance patients’ sense of safety?

II – Living with Cognitive Consequences

How should support be designed to help when life is affected by cognitive difficulties?

How can we provide support for managing fatigue?

How can we support patients in dealing with impaired executive function, lack of concentration, and memory problems?

How can we support patients in managing behavioral changes?

How can we encourage and facilitate activity and social interaction?

III – Receiving Individualized Care

How can we meet patients and their relatives as unique and important individuals?

How can we communicate based on factual information?

For patients without close relatives – how can we tailor support to their needs?

How can we make use of technological solutions?

IV – A Life in Uncertainty

Uncertainty about treatment effectiveness

Uncertainty about survival time

Uncertainty about the future in both short- and long-term perspectives

Uncertainty about symptom progression

How can we provide support to reduce patients’ and relatives’ experience of uncertainty? Any good examples?

V – Emotional and Existential Support

How can we help families address emotional and existential issues/distress?

When and how should support be provided?

How can we encourage families to seek help from their social networks?

VI – Managing the New Life Situation

How do we help patients and relatives prioritize what they find most important in life?

How do we encourage continued engagement in leisure activities, travel, spending time with family and friends, etc.?

How do we help manage physical and cognitive symptoms?

How do we encourage families to plan and talk about illness and the future together?

VII – Breaking Isolation

How can we help break isolation?

Closing Question

Is there anything else you think is important regarding how support can be developed for patients and their relatives, based on the care pathway?

1. **Supplementary tables**

**Supplementary table 1.** Selection of participant quotes from the category *Organization and structural decisions* grouped by subcategory.

| **Subcategory** |  | **Participant quote** |
| --- | --- | --- |
| *Proactive planning and timely support* |  | **#1**  “But I think that it is, because you asked before, like this, we have a structure for these, for all that we can do and know. And I think that none of us has like a manual that we carry with us (…) so that’s why I think that we have like, there is an inner…” (Social worker, regional healthcare)  “Structure.” (Social worker, regional healthcare)  **#2**  “I was talking about plan A and plan B (…) Plan A is life. Plan B is if things go wrong. But then you’ve made this plan A, you’ve written where you want to be buried, and who’s going to get your money and who’s going to get that nice painting, and all that. And then you put it away in a drawer and close it, and then you run plan A, until you need to use plan B (…)” (Social worker, regional healthcare)  “There are certain cases where it goes very, very quickly. Then they might not have had time to do it. (..), and then, then it might be too late for the patient to arrange this as well.” (Social worker, regional healthcare) |
| *Integrated care pathways* |  | **#1**  "Then it's, it's also a very quick process, uh, from investigation to surgery. It's a process for highly malignant cases that takes a few weeks. So it's firstly, firstly there after surgery, but then, then comes the next phase during treatment, and then it's, then we hand them over (…) during the treatment period except for certain parts, but that can be a period where we don't have much contact with the patients." (Specialist Nurse, regional healthcare)  **#2**  "Yes, yes absolutely, and we have them all the time. So it's not that they're not allowed to call us, they're welcome to call and they do, because we're easily accessible." (Specialist Nurse, regional healthcare)  **#3**  "I also think that there may be certain periods where they may have a little more frequent contact with us as social workers precisely because we need to adjust the approved interventions, um, but then it is also that they, it is good if they make that contact or if we receive the information from home care or healthcare that now there is a changed need, it looked different six months ago, now we need to adjust. That is what you have to do." (Social worker, municipal services)  **#4**  "(…) so that if you had already had that contact (Palliative Resource Team) then more patients would have said okay, we're stopping now, but if you say we're stopping now and then we won't take care of you anymore, but I'll send you over to another team, then it feels much harder for them to stop." (Specialist physician, regional healthcare) |
|  |  |  |

**Supplementary table 2.** Selection of participant quotes from the category *Interactions and interpersonal dynamics* grouped by subcategory.

| **Subcategory** |  | **Participant quote** |
| --- | --- | --- |
| *Psychosocial and personal circumstances* |  | **#1**  “(…) that fear of the future, it is immense, immense; you see it a lot. Um, it comes, the patients who come to us, they already have a diagnosis. (…). And there is that fear of not knowing how long they have left. What will happen. Um, how will the disease progress. How quickly it progresses. How the process goes, how quickly it goes.” (Specialist physician, regional healthcare)  **#2**  “But we see that it is needed, lots of different aids, and they just often say that no, it can’t be done, we don’t want that, the home is invaded, we want it to be as usual, it’s going well, it’s, no, even though they are sick they say very many times, well, it’s going well, the family helps me. We can manage.” (Nurse^a^, municipal healthcare)  “There are many who oppose, for example, a nursing bed like that. Then the home looks like a hospital (…)” (Nurse^a^, municipal healthcare)  **#3**  “(…) relatives need to take that part or replace it, or whatever you want to say, for everyday life to function so that it is…” (Specialist nurse, regional healthcare)  “It is a huge role.” (Social worker, regional healthcare); “It is a huge role and burden (…)” (Specialist nurse, regional healthcare) |
| *Expectations and misalignments* |  | **#1**  "I think, I think it's important and I also think that our doctors are very good at it, at being honest. (…) They (patients) are more prepared for deterioration if they know that it's a bad prognosis, so I think that we and/or the doctors are very good at being honest with the prognosis and saying it like it is, but still when you leave the clinic, you leave with a feeling that you've had a good conversation with them". (Specialist nurse, regional healthcare)  **#2**  “And no one has really asked the relative if this is what you want to be?” (Specialist nurse, regional healthcare)  “Are you taking this on now?” (Social worker, regional healthcare)  “No, it's not, but you have become, when the patient has received their diagnosis, then you are called to the hospital then, to be involved, then you are not allowed to be a patient, as a relative, but you have to take care of this somewhere else.” (Specialist nurse, regional healthcare) |
| *Dialogue and affirmation* |  | **#1**  “And then also that we, I think that we as fellow human beings can face them in those issues as well, and we do.” (Other members of the multidisciplinary team^b^, regional healthcare)  **#2**  “Exactly. Yes, I think that it’s like, I can talk to patients about their faith and doubts or hopes related to what the Quran says I should do and what the doctor says I should do. It’s, as long as you do it, as long as the patient wants you to do it so to speak or, there’s no obstacle for that. Um. Yes.” (Specialist Nurse, regional healthcare)  **#3**  “(…) it doesn’t just apply to this patient group, but many people get their binder when they come to the doctor’s appointment where it says fatigue and it says a lot about nausea and stomach and intestinal problems and similar issues, but if you – it’s not enough – it’s unusual, in my experience anyway, that they just… sort of say, yes, but I’ve put that in my cancer bag.” (Other members of the multidisciplinary team^b^ healthcare) |
|  |  |  |

^a^ To enhance confidentiality, specialist and non-specialist nurses in the municipal healthcare are disclosed as “Nurse”.

^b^ To enhance confidentiality, physiotherapists, occupational therapists, neuropsychologists, nutritionists, and speech therapists are disclosed together as “Other members of the multidisciplinary team”.
